# Effect of Probiotics on Liver Enzymes in Patients With Non-alcoholic Fatty Liver Disease: An Umbrella of Systematic Review and Meta-Analysis

**DOI:** 10.3389/fnut.2022.844242

**Published:** 2022-05-23

**Authors:** Vali Musazadeh, Neda Roshanravan, Parvin Dehghan, Sana Sedgh Ahrabi

**Affiliations:** ^1^Student Research Committee, Tabriz University of Medical Sciences, Tabriz, Iran; ^2^Department of Community Nutrition, School of Nutrition and Food Science, Tabriz University of Medical Sciences, Tabriz, Iran; ^3^Cardiovascular Research Center, Tabriz University of Medical Sciences, Tabriz, Iran; ^4^Faculty of Nutrition and Food Science, Nutrition Research Center, Tabriz University of Medical Sciences, Tabriz, Iran; ^5^Immunology Research Center, Tabriz University of Medical Sciences, Tabriz, Iran

**Keywords:** non-alcoholic fatty liver disease, probiotics, liver enzyme, umbrella meta-analysis, systematic review

## Abstract

Non-alcoholic fatty liver disease (NAFLD) has become prevalent in recent decades, especially in developed countries; yet the approaches for preventing and treating NAFLD are not clear. This study aimed to summarize meta-analyses of randomized controlled trials that examined the effects of probiotics on NAFLD. We systematically searched PubMed, Scopus, Embase, Web of Science, and Cochrane Central Library databases up to August 2021. All Meta-analysis studies assessing the effect of probiotics on liver function tests [alanine aminotransferase (ALT), aspartate aminotransferase (AST), and Gamma-glutamyl transferase (GGT)] were included. Meta-analysis was conducted using a random-effects model. Sensitivity and subgroup analyses were also performed. The umbrella study covered ten eligible studies involving 5,162 individuals. Beneficial effects of probiotics supplementation were revealed on ALT (ES = −10.54 IU/L; 95% CI: −12.70, −8.39; *p* < 0.001; *I*^2^ = 60.9%, *p* = 0.006), AST (ES = −10.19 IU/L, 95%CI: −13.08, −7.29, *p* < 0.001; *I*^2^ = 79.8%, *p* < 0.001), and GGT (ES = −5.88 IU/L, 95% CI: −7.09, −4.67, *p* = 0.009; *I*^2^ = 0.0%, *p* = 0.591) levels. Probiotics have ameliorating effects on ALT, AST, and GGT levels in patients with NAFLD. Overall, Probiotics could be recommended as an adjuvant therapeutic method for the management of NAFLD.

## Introduction

Non-alcoholic fatty liver disease (NAFLD) is characterized by lipid deposition in liver cells (1). On the other hand, untreated NAFLD can cause non-alcoholic hepatitis (NASH) as well as other liver-related severe sicknesses like cirrhosis, liver failure, and liver cancer (2). Considering the newest epidemiological studies, the estimated prevalence of NAFLD in Middle Eastern countries is 31.8%, the highest in comparison with other regions in the world (3). Also, studies display a higher prevalence of NAFLD in patients having obesity or hyperlipidemia (4). NAFLD has a strong relationship with metabolic syndromes like obesity, insulin resistance, type 2 diabetes, dyslipidemia, and hypertension (1, 5–8). Besides liver, this disease can progress and affect many other organs, such as systemic arteries, heart and kidneys, and causes irreversible problems and death (7). It has been suggested that lifestyle changes such as diet and exercise interventions, can help manage NAFLD and lead to a decrease in occurrence and progression of NAFLD (6, 7, 9). Therefore, studies showed that nutritional changes and exercise could lower liver lipid, improve liver enzyme functions and reduce plasma triglyceride (10, 11). However, earlier studies reported that natural compounds can reduce the complications caused by NAFLD (1, 12).

Some studies have shown that there is an association between the gut-liver axis and NAFLD. More than 10,000 microbiomes live in a synergetic relationship with the intestinal tract and have different effects on the host’s health and disease condition (13–15). Recently, microbiome-targeted treatments (MTTs) are a proposed way to influence the gut microbiome. In this regard, probiotics are used as a treatment strategy. Probiotics are explained as live microbial organisms used as dietary supplements that are beneficial for the human or animal host in terms of health. Moreover, when provided in an adequate dosage and for a capable duration, probiotics can improve intestinal microbial balance (16) and cause disease occurrence to delay by balancing intestinal flora, permeability, and inflammations (17). Improved production of short-chain fatty acids such as butyrate through microbial pathways impacts the metabolism of energy in the intestine and overall body (18, 19). In addition, probiotics can develop anti-microbial compounds and acidity of the intestinal lumen and cause a reduction in generation of pathogens (20). Finally, a variety of microorganism-based products can impact the immunity of the host positively (21, 22).

Although, many accumulative evidences have shown the beneficial effects of probiotics on liver enzymes by affecting specific biological processes; however, there are some contradictions in this regard (23–27). For more definite results, we aimed to investigate the effects of probiotics supplementation on serum levels of alanine transaminase (ALT), AST, and GGT in an umbrella meta-analysis study.

## Methods

### Search Strategy and Study Selection

We followed standardized methods to carry out this umbrella review (a systematic review of multiple meta-analyses) to provide a clear understanding in terms of probiotics supplementation and serum levels of ALT, AST, and GGT. The scientific international databases including, PubMed, Scopus, EMBASE, Web of Science, and Cochrane Central Library databases were searched for relevant studies published up to August 2021. The pattern of search strategy is provided in Supplementary Table 1. To enhance the sensitivity of our search strategy, the wild-card term “*” was used. English-language articles were included in this study.

### Inclusion and Exclusion Criteria

Meta-analysis studies examining the effects of probiotics supplementation on liver enzymes (ALT, AST, and GGT) which reported the effect sizes (ES) and corresponding confidence intervals (CI), were included in the umbrella meta-analysis. Additionally, we excluded the following studies: *in vitro*, *in vivo*, and *ex-vivo* studies, case reports, observational studies, quasi-experimental studies, and controlled clinical trials.

### Quality Evaluation

The methodological quality of meta-analyses was evaluated by two reviewers (VM and SSA) independently using the AMSTAR questionnaire (28). The AMSTAR questionnaire contains 11 items that asks reviewers to answer “Yes,” “No,” “Can’t answer” or “Not applicable.” The maximum score is 11. Articles with scores higher than seven are considered as high-quality studies.

### Study Selection and Data Extraction

Two independent reviewers (VM and SSA) screened the articles based on mentioned eligibility criteria. After checking and excluding irrelevant studies by the titles and abstracts, the full text of the relevant articles was evaluated to identify the study’s eligibility for the umbrella meta-analysis. Any disagreement was resolved through the consensus with the third author (PD).

The first authors’ name, year of publication, sample size, study location, dosage, and duration range of supplementation, ESs and CIs for ALT, AST, and GGT were extracted from the selected meta-analyses.

### Data Synthesis and Statistical Analysis

ESs and CIs were used to estimate the overall effect sizes. Heterogeneity was determined by *I*^2^ statistics and Cochrane’s *Q*-test. *I*^2^-value > 50% or *p* < 0.1 for the *Q*-test was considered as significant between-study heterogeneity. A random-effects model was applied to perform meta-analysis when the between-study heterogeneity was significant; otherwise, the fixed-effects model was employed. To detect probable sources of heterogeneity, subgroup analyses were performed according to the predefined variables, including type of effect size, study duration, mean age, sample size, and study location. Sensitivity analysis was used to identify the dependency of the overall effect size on a special study. The Egger’s and Begg’s tests was performed to detect a small-study effect. Publication bias was identified by visual inspection of the funnel plot. If there was any evidence of publication bias or small-study effect, trim and fill analysis was carried out accordingly. The meta-analysis was done using Stata, version 16 (Stata Corporation, College Station, TX, US). *P*-value < 0.05 was considered as significance level.

## Results

### Study Selection and Study Characteristics

**FIGURE 1:**
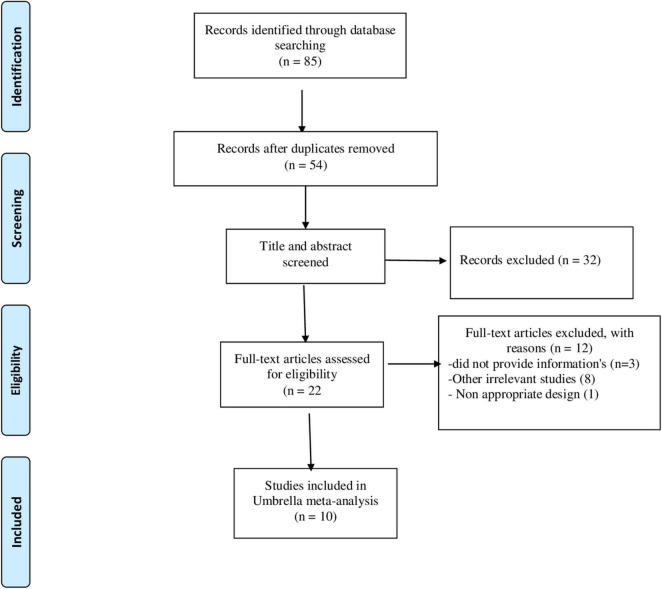
Flow diagram of study selection.

A total number of 85 articles were identified through a systematic search of electronic databases. Putting aside the 31 duplicate articles, 54 articles were screened carefully by titles and abstracts, among which 22 articles were selected for consideration by full-text evaluation. Considering the inclusion criteria, ten articles were included in the umbrella meta-analysis. Figure 1 presented the flow diagram of the study selection process. According to the studied variables, the distribution of identified articles was ten articles for ALT, nine for AST, and three for GGT. The included studies were conducted between 2013 and 2019, and the mean age of participants was 44 years. The average dose of probiotics in studies was between 2.6 × 10^9^ and 5 × 10^11^ CFU. Studies were fulfilled in China (23, 25, 26, 29, 30), United States (31–33), India (34), and France (27). The duration of studies ranged between 8 and 20 weeks. Cochrane Risk of Bias Tool (35), adapted from Littell et al. (36), Physiotherapy Evidence Database (PEDro) scale tool (37) and Jadad scores (38) were used for quality assessment. Overall, almost 90% of meta-analyses included high quality RCTs. The quality of the RCTs included in the meta-analyses is summarized in Table 1.

**TABLE 1:** Study characteristics of included studies.

References	No. of studies in meta-analysis	Location duration (week)	No. of participants in meta-analysis	Age (year)	Dose (mg/day)	Quality assessment scale and outcome
Gao et al. (23)	9	China 16 week	268	37	*Bifidobacterium*, *Lactobacillus*, *Streptococcus*, VSL#3 NR	Yes (Cochrane) 9/9 high
Khan et al. (32)	12	United States 17 week	292	50	*Lactobacillus*, *Bifidobacterium*, probiotics yogurt 11.4*10^9^	Yes (Cochrane) 9/13 high
Loman et al. (31)	11	United States**** 8 week	195	34	*Lactobacillus*, *Bifidobacterium*, *Streptococccus* 2.6*109	Yes (adapted from Littell et al.) 7/11 high
Sharpton et al. (33)	16	United States 10 week	322	NR	*Lactobacillus*, *Bifidobacterium*, probiotics yogurt NR	Yes (Cochrane) 11/16 high
Liu et al. (29)	15	China 11 week	673	37	*Acetobacter*, *Bifidobacterium*, *Lactobacillus*, *Streptococcus*	Yes (Cochrane) 13/15 high
Lavekar et al. (34)	7	India 19.5 week	138	NR	*Lactobacillus* NR	Yes (Jodad) 7/7 high
Tang et al. (26)	22	China 13.5 week	879	30	*Lactobacillus*, *Bifidobacterium*, *Streptococccus*, *Bacillus*, *Enterococcus* NR	Yes (Cochrane) 20/22 high
Xiao et al. (25)	28	China 16 week	420	40	*Lactobacillus*, *Streptococcus*, *Bifidobacterium*, *Propionibacterium*, *Acetobacter* NR	Yes (Cochrane) 20/28 high
Koutnikova et al. (27)	99	France 8 week	1971	NR	*Bifidobacteria*, *Streprococcus*, *Salivarius*, *Lactobacilli* 5 × 10^11^	Yes (PEDro scale tool) 84/99 high
Ma et al. (30)	4	China 17 week	268	44	*Lactobacillus*, *Streptococcus*, *Bifidobacterium*, *Lepicol probiotic* NR	Yes (Jodad) 4/4 high

*PEDro, Physiotherapy Evidence Database scale tool.*

### Methodological Quality

AMSTAR checklist assessments for the methodological quality of the covered studies are summarized in Table 2. All meta-analyses included in the umbrella meta-analysis had a high-quality.

**TABLE 2:** Detailed evaluation of the methodological quality with AMSTAR^a^.

Study	Q1^b^	Q2	Q3	Q4	Q5	Q6	Q7	Q8	Q9	Q10	Q11	Quality score
Khan et al. (32)	Yes	Yes	Yes	Yes	Yes	NO	Yes	NO	Yes	NO	Yes	8
Lavekar et al. (34)	Yes	CA	NO	Yes	NO	Yes	Yes	NO	Yes	CA	NO	5
Xiao et al. (25)	Yes	Yes	Yes	Yes	Yes	Yes	Yes	Yes	Yes	Yes	Yes	11
Liu et al. (29)	Yes	Yes	Yes	Yes	Yes	Yes	Yes	Yes	Yes	NO	Yes	10
Loman et al. (31)	Yes	Yes	Yes	Yes	Yes	Yes	Yes	Yes	Yes	Yes	Yes	11
Ma et al. (30)	Yes	Yes	Yes	Yes	Yes	Yes	Yes	NO	Yes	NO	NO	8
Sharpton et al. (33)	Yes	Yes	Yes	Yes	Yes	CA	Yes	NO	Yes	Yes	NO	8
Tang et al. (26)	Yes	Yes	Yes	Yes	Yes	Yes	Yes	Yes	Yes	Yes	Yes	11
Gao et al. (23)	Yes	Yes	Yes	Yes	Yes	Yes	Yes	NO	NO	NO	NO	7

*^a^AMSTAR, assessment of multiple systematic reviews; CA, can’t answer; Q, Question.*

*^b^Q1, Was an “a priori” design provided?; Q2, Was there duplicate study selection and data extraction?; Q3, Was a comprehensive literature search (at least two databases) performed?; Q4, Was the status of publication (i.e., gray literature) used as an inclusion criterion?; Q5, Was a list of studies (included and excluded) provided?; Q6, Were the characteristics of the included studies provided?; Q7, Was the scientific quality of the included studies assessed and documented?; Q8, Was the scientific quality of the included studies used appropriately in formulation conclusions?; Q9, Were the methods used to combine the findings of studies appropriate?; Q10, Was the likelihood of publication bias assessed?; Q11, Was the conflict of interest included?*

### Effect of Probiotics on Alanine Aminotransferase

**FIGURE 2:**
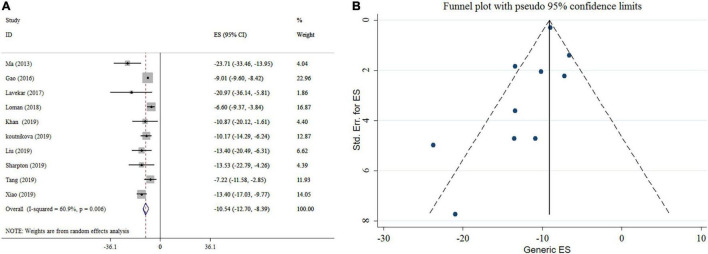
Forest plot **(A)** detailing mean difference and 95% confidence intervals (CIs) and funnel plot **(B)** displaying publication bias in the studies reporting, the effects of probiotics supplementation on ALT levels.

There was a significant reducing impact of probiotics on ALT (Figure 2A). Notable heterogeneity was observed among studies (*I*^2^ = 60.9%, *p* = 0.006). The high heterogeneity was reduced after subgroup analysis based on the type of effect size, sample size, study location, and duration of intervention. Reductions in ALT levels were more pronounced in the subgroups of sample size (<300) and an intervention duration ≥ 16 weeks when compared to their counterparts (Table 3). The sensitivity analysis revealed that the calculated overall effect sizes for ALT were not significantly changed after omitting each study. No significant small-study effects was found using Egger’s and Begg’s tests (*p* = 0.1 and *p* = 0.074, respectively). Moreover, visual inspection of the funnel plot indicated an asymmetric distribution of studies (Figure 2B). Thus, trim and fill analysis was conducted with ten studies (none imputed studies). The corrected effect size for publication bias didn’t show any change after trim and fill analysis.

**TABLE 3:** Pooled estimates of probiotics effects on liver enzymes within different subgroups.

Variables	No. study	Pooled effect size (95% CI)	*p*-value	I^2^ (%)	P heterogeneity
**ALT** Total **Type of effect size** WMD *MD*	10 5 5	−10.54 (−12.70, −8.39) −11.61 (−15.24, −7.97) −9.91 (−13.35, −6.46)	<0.001 <0.001 <0.001	60.9 74.4 44.0	0.006 0.004 0.129
**Sample size**<300 ≥300	5 5	−13.77 (−20.49, −7.05) −9.96 (−11.98, −7.93)	<0.001 <0.001	74.5 45.4	0.004 0.119
**Age (years)**					
≤45	3	−13.89 (−22.66, −5.13)	0.002	79.7	0.007
>45	3	−8.47 (−10.14, −6.80)	<0.001	32.5	0.227
NR	4	−12.33 (−14.88, −9.77)	<0.001	0.0	0.432
**Intervention duration (week)**					
<16 ≥16	5 5	−8.76 (−11.25, −6.28) −13.50 (−18.18, −8.81)	<0.001 <0.001	30.2 75.7	0.22 0.002
**Country**					
China Other	5 5	−11.66 (−15.21, −8.21) −9.62 (−13.00, −6.23)	<0.001 <0.001	75.3 38.0	0.003 0.168
**AST** Total **Type of effect size** WMD MD	9 4 5	−10.19 (−13.08, −7.29) −10.37 (−13.66, −7.07) −10.58 (−16.13, −5.02)	<0.001 <0.001 <0.001	79.8 59.4 82.0	<0.001 0.06 <0.001
**Age (years)**					
≤ 45	3	−11.51 (−17.80, −5.23)	<0.001	52.6	0.121
> 45	3	−7.25 (−11.98, −2.52)	<0.001	91.2	<0.001
NR	4	−12.85 (−17.00, −8.70)	<0.001	44.7	0.164
**Intervention duration (week)**					
<16 ≥16	4 5	−7.79 (−12.10, −3.49) −12.89 (−17.13, −8.66)	<0.001 <0.001	77.0 64.7	0.005 0.023
**Country**					
China Other	5 4	−10.65 (−13.58, −7.71) −10.02 (−16.24, −3.79)	<0.001 0.002	53.3 83.4	0.073 <0.001

### Effect of Probiotics on Aspartate Aminotransferase

**FIGURE 3:**
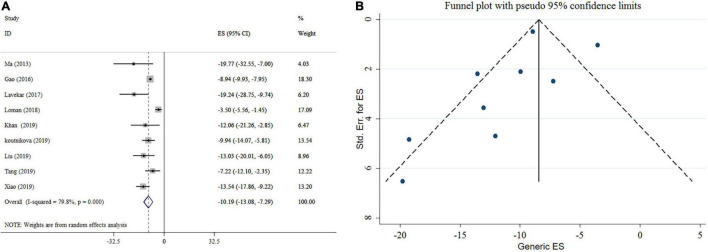
Forest plot **(A)** detailing mean difference and 95% confidence intervals (CIs) and funnel plot **(B)** displaying publication bias in the studies reporting, the effects of probiotics supplementation on AST levels.

Probiotics supplementation significantly reduced AST level according to the meta-analysis of nine studies (Figure 3A). Heterogeneity was observed between studies (*I*^2^ = 79.8%, *p* < 0.001). For AST; pooled analysis, type of effect size, sample size, study location, and duration of intervention were possible sources of heterogeneity. Subgroup analysis indicated that probiotics supplementation with an intervention duration ≥ 16 weeks and a sample size of < 300 contributes to a more significant effect in lowering AST (Table 3). Sensitivity analysis revealed that no single study likely affected the pooled results. No significant small-study effect was shown using Egger’s and Begg’s tests (*p* = 0.318 and 0.466, respectively). Visual inspection of the funnel plot demonstrated a significant publication bias among included studies (Figure 3B). In the following trim and fill analysis, the effect size did not alter.

### Effect of Probiotics on Gamma-Glutamyl Transferase

**FIGURE 4:**
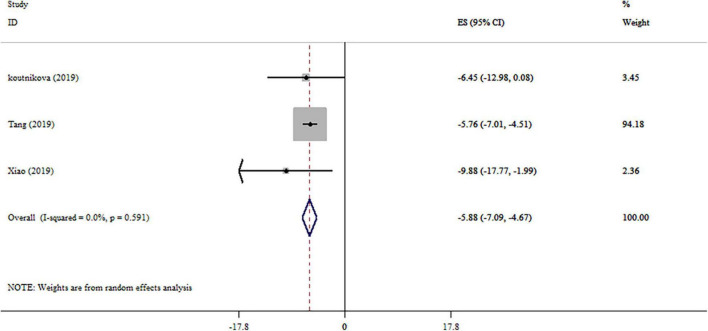
Forest plot detailing mean difference and 95% confidence intervals (CIs), the effects of probiotics supplementation on GGT levels.

The effect of probiotics on GGT level was reported in three studies (Figure 4). Our analysis revealed a significant reduction in GGT levels by probiotics intervention. No remarkable heterogeneity was observed between studies (*I*^2^ = 0.0%, *p* = 0.591). Subgroup analysis wasn’t conducted on studies. Sensitivity analysis provided no evidence of the impact of an individual study on the overall effect size. After performing the Begg’s tests, no small-study effects was detected (*p* = 0.296).

## Discussion

Exposure to probiotics has been the subject of numerous meta-analyses in the realm of health as well as a diverse range of disorders. We conducted this umbrella review to summarize the available evidence and draw conclusions for the effects of probiotics in NAFLD treatment. We identified 10 meta-analyses of RCTs which had high quality based on the AMSTAR checklist. There seem to be beneficial associations between probiotic consumption and liver function in terms of serum ALT, AST, and GGT levels. All-inclusive, probiotic therapy generally appears to be safe without any evidence of adverse effects.

Even though the findings suggest that probiotics supplementation may be efficacious for controlling NAFLD, it must be stated that, the effects of probiotics on liver enzymes (ALT & AST) were heterogeneous. Differences in treatment dosage, sample size, gender, and duration of intervention, mean age of participants, study location, and population may explain this heterogeneity. Also, the evidence from this study implies that probiotic supplementation in studies with sample size < 300 and supplement duration ≥ 16 weeks can meaningfully decrease ALT. In line with ALT reduction, probiotic supplementation with an intervention duration ≥ 16 weeks and a sample size of < 300 contributes to a more significant effect in lowering AST. Also, probiotic consumption had beneficial effect on reducing GGT level without any significant heterogeneity (39, 40).

NAFLD is a chronic liver disease with a worldwide prevalence of 20–30%. It can be attributed to numerous roots, including environmental parameters, metabolic, genetic, and gut microbial factors (41). In the past decade, considerable evidence has been accumulated regarding the critical role of gut microbiota unbalance and various metabolic disorders, including NAFLD. The causal role of gut microbiota dysbiosis (an imbalance in microbial homeostasis) in NAFLD genesis and development has been reported (42, 43). Host physiology, age, drugs like antibiotics, diet, and environmental factors can influence the gut microbial ecosystem (44).

The gut dysbiosis may influence the development of NAFLD via various signaling pathways including (45, 46):

• Impact on intestinal hormone production affecting glucose control.
• Effect on short-chain production affecting glucose and lipid metabolism.
• Increasing liver toxicity and cardiovascular risk.
• Disturbance of bile acid homeostasis.
• Contribution in metabolic endotoxemia via bacterial lipopolysaccharide (LPS) which subsequently triggers intestinal and hepatic inflammation.

In addition, gut dysbiosis cause the intestinal more permeable which leading to an elevation in fatty acid absorption, migration of the bacteria via the gut epithelial barrier, release of toxic bacterial products and pro- inflammatory cytokines that can initiate an inflammatory cascade. Interestingly, recent studies indicated that gut dysbiosis is related to higher fecal concentrations of some metabolites (2-butanone and 4-methyl-2-pentanone) which lead to hepatocellular toxicity (47).

Probiotics are believed to be important for liver health via specific biological processes as follows: (1) increment in insulin sensitivity; (2) decreasing the absorption of glucose and LDL cholesterol; (3) modification of gut dysbiosis and inducing the production of short-chain fatty acids; (4) decrement in endotoxin concentrations; (5) reducing the oxidative stress status and inflammatory markers; (6) diminishing total cholesterol levels and (7) trapping of bile acids’ substances (48–51). Interestingly, probiotics have been introduced as an effective agent in inhibiting pathogens. The fermented products of many probiotic bacteria, containing lactic and acetic acids, are significant causes of probiotics’ antimicrobial features. Furthermore, bacteriocins (small antimicrobial proteins secreted by some probiotics) are another cause for this trait (52).

The overall evidence indicates that supplementation with probiotics led to a reduced level of liver enzymes (53). ALT, AST, and GGT are often used to indicate the quality of liver function. The correlation of the entitled enzymes with NAFLD has been shown in some previous studies (54, 55). In recent prospective studies, GGT is considered as a sensitive indicator of liver damage and a novel marker for oxidative stress as well as for inflammation (56, 57). In addition, there is an association between liver aminotransferase enzymes’ concentration (AST and ALT) and the quality of liver function (58, 59). Based on previous reports, increased ALT levels in patients with NAFLD may be related to insulin resistance and intrahepatic fat content (60). Lower AST and ALT levels were perceptible in various meta-analyses following probiotics supplementation (25–27, 29–34). Beyond that, microbial therapies reduced circulating GGT according to other evidence-based meta-analyses (25, 27, 31). Several probiotic strains have particular abilities to improve liver function through the modulation of the gastrointestinal tract. These improvements were mostly observed with *Bifidobacterium*, *Lactobacillus*, and *Streptococcus* or multispecies probiotic therapy (23, 27). Studies specifically analyzing the gut microbiome composition revealed that the imbalanced gut–liver axis can be a major factor in NAFLD development and progression (61). In general, the potential therapeutic effects of the gut microbiome manipulation by probiotics and changes in the relative abundance of selective bacteria can be considered as an alternative therapy in NAFLD patients.

## Strengths and Limitations

This umbrella review used systematic methods with robust search strategies of various databases and independent study selection and extraction methods, which systematically summarized the current evidence regarding the effects of probiotics supplementation on serum levels of ALT, AST, and GGT. Moreover, the quality of covered systematic reviews and meta-analyses were evaluated using the AMSTAR questioner. However, the use of existing meta-analyses is the main limitation of the umbrella review. The results may depend on what assessments to choose from each preliminary study and how to report them in the meta-analysis. Finally, the most critical limitation of this study is to consider that an impressive body of pioneering studies has supported the concept that in the absence of aminotransferase levels abnormalities, the NAFLD may pretend (62–64). So in fact these criteria may not be reliable for the diagnosis of NAFLD.

## Conclusion

The beneficial impacts of probiotics as new promising therapeutic agents for patients with NAFLD have been summarized in this studies. Convincing evidence was obtained regarding associations between probiotic intake and liver function improvement reflecting as the serum levels of ALT, AST, and GGT.

However, due to the high heterogeneity of the results and the small number of studies included in each subgroup, the outcomes of the present study should be interpreted with caution.

Even though most studies showed hepatic enzymes as biomarkers for liver function, there is still no comprehensive agreement on counting on the enzyme levels in order to indicate liver function. Adding other non-invasive assessments in line with enzyme levels in RCTs seems to be a necessity. Conclusively, further studies need to be conducted for a definite presumption.

## Data Availability Statement

The original contributions presented in the study are included in the article/Supplementary Material, further inquiries can be directed to the corresponding author/s. All the materials used in this systematic review and meta-analysis have been fully referenced.

## Author Contributions

VM and SA wrote the original manuscript and contributed to the conception of the article. VM and NR contributed to data collection and analyze and provided advice and consultation. PD contributed to the final revision of the manuscript. All authors read and approved the final manuscript.

## Conflict of Interest

The authors declare that the research was conducted in the absence of any commercial or financial relationships that could be construed as a potential conflict of interest.

## Publisher’s Note

All claims expressed in this article are solely those of the authors and do not necessarily represent those of their affiliated organizations, or those of the publisher, the editors and the reviewers. Any product that may be evaluated in this article, or claim that may be made by its manufacturer, is not guaranteed or endorsed by the publisher.

## Supplementary Material

Supplementary Table 1 Electronic search strategy.

## Abbreviations

NAFLD: Non-alcoholic fatty liver disease
ALT: Alanine aminotransferase
AST: Aspartate aminotransferase
GGT: Gamma-glutamyl transferase
NASH: Non-alcoholic hepatitis
MTTs: microbiome-targeted treatments
ES: effect sizes
CI: corresponding confidence intervals.
